# Germline copy number variations are associated with breast cancer risk and prognosis

**DOI:** 10.1038/s41598-017-14799-7

**Published:** 2017-11-07

**Authors:** Mahalakshmi Kumaran, Carol E. Cass, Kathryn Graham, John R. Mackey, Roland Hubaux, Wan Lam, Yutaka Yasui, Sambasivarao Damaraju

**Affiliations:** 1 Department of Laboratory Medicine and Pathology, University of Alberta, Edmonton, AB Canada; 2 Department of Oncology, University of Alberta, Edmonton, Alberta Canada; 3 Department of Integrative Oncology, British Columbia Cancer Agency, Vancouver, BC Canada; 4 School of Public Health, University of Alberta, Edmonton, Alberta Canada; 5 Cross Cancer Institute, Alberta Health Services, Edmonton, AB Canada

## Abstract

Breast cancer is one of the most common cancers among women, and susceptibility is explained by genetic, lifestyle and environmental components. Copy Number Variants (CNVs) are structural DNA variations that contribute to diverse phenotypes via gene-dosage effects or cis-regulation. In this study, we aimed to identify germline CNVs associated with breast cancer susceptibility and their relevance to prognosis. We performed whole genome CNV genotyping in 422 cases and 348 controls using Human Affymetrix SNP 6 array. Principal component analysis for population stratification revealed 84 outliers leaving 366 cases and 320 controls of Caucasian ancestry for association analysis; CNVs with frequency > 10% and overlapping with protein coding genes were considered for breast cancer risk and prognostic relevance. Coding genes within the CNVs identified were interrogated for gene- dosage effects by correlating copy number status with gene expression profiles in breast tumor tissue. We identified 200 CNVs associated with breast cancer (q-value < 0.05). Of these, 21 CNV regions (overlapping with 22 genes) also showed association with prognosis. We validated representative CNVs overlapping with *APOBEC3B* and *GSTM*1 genes using the TaqMan assay. Germline CNVs conferred dosage effects on gene expression in breast tissue. The candidate CNVs identified in this study warrant independent replication.

## Introduction

Breast Cancer is one of the commonly diagnosed cancers among women worldwide^1^, in Canada, breast cancer accounts for about 25% of all diagnosed cancers, and 15% of all cancer deaths^2^. Based on twin studies, estimated heritable genetic factors contribute to about 30% for breast cancer risk, the remaining risk being due to environmental and lifestyle factors^3^. Family based linkage and genome sequencing studies have identified high and moderate penetrant mutations in genes such as *BRCA 1* or *BRCA 2* ^4,5^ *PTEN* ^6^, *PALB2* ^7^, *ATM* ^8^, *TP53* ^9^, and *CHECK2* ^10^ that contribute to the genetic risk of breast cancers. Subsequently, large scale population based Genome Wide Association Studies (GWAS) were successful in identifying several low penetrant common genetic variants (Single Nucleotide Polymorphisms, SNPs) associated with breast cancer risk. Among these, a limited number of GWAS SNPs (7 SNPs) showed effect sizes (odds ratio or ORs) between 1.25–1.5 and the remaining SNPs showed effect sizes < 1.25^11,12^. SNP based GWAS served as a valuable tool in uncovering novel genes or loci associated with breast cancer aetiology. Low, moderate and high penetrant SNPs and mutations together explain up to 50% of the genetic risk associated with breast cancer^11,12^, and the remaining variants to explain the “missing heritability” are yet to be discovered. Copy Number Variations (CNVs) in the germline DNA are currently being investigated to explain missing heritable risk for breast cancer^13^.

Germline CNVs are a class of structural variations, and are defined as loss or gain of genomic DNA in size range of 50 bp to 1 Mb^14^. Germline CNVs are studied as genetic determinants for susceptibility for familial breast cancer^15–20^ and also cancers of prostate^21–23^, ovary^18,24–26^, pancreas^27–29^, colon, rectum^16,30–34^, endometrium^35^, lung^36–38^ and melanoma^39,40^.

The DNA sequence coverage for CNVs is ~10% of the genome. CNVs harbour coding regions and non-coding regulatory regions and may confer profound phenotypic effects relative to effects caused by SNPs^41–43^. CNVs have a multitude of effects based on their genomic location including gene dosage effects and *cis*-regulatory functions^23^. Since the distribution of CNVs across the genome is disproportionate with a higher proportion in non-coding than coding regions, their functional impact on phenotype is not clear. However, CNVs that overlap protein coding genes offer insights into disease phenotypes and associated biology^44^. Nearly 80% of cancer genes harbour CNVs^45^ and support the above premise.

The majority of the CNVs that have been identified to-date for breast cancer are rare (frequency < 1%) and may potentially confer high penetrance (odds ratios > 3.0) in familial breast cancer^18,20^. Associations of low penetrant common CNVs identified using GWAS have been shown in prostate^21,22^ and pancreatic^29^ cancers. CNV-GWAS has met with considerable success in several complex disease phenotypes^46^ but is lagging in breast cancer with a limited number of studies adopting this approach. Long *et al*. in 2013 was the first to report a common CNV (deletion) in a coding gene using GWAS, wherein *APOBEC3* loci were shown to be associated with breast cancer risk in a Chinese population^47^. This deletion polymorphism was also validated in a Caucasian population^48^. These results support the goal of searching for common germline CNVs associated with sporadic breast cancer to address missing heritability in populations. This is in contrast to earlier claims that common CNVs were not associated with breast cancer^49^.

Tumor based markers for prognosis are useful in guiding treatments but markers with higher specificity are needed to account for inter-individual variations in breast cancer prognosis. DNA level aberrations (CNVs) from tumor (somatic) genomes were shown to be prognostic. However, such studies do not distinguish origins from germline CNVs or de novo copy number aberrations in somatic cells due to genomic instability. Our current emphasis is to assess the role of germline copy number variations for their prognostic value. SNPs showing association with breast cancer susceptibility were not prognostic^50,51^. Because independent SNP based GWAS for prognosis in breast cancer were not informative^2,50–53^, we focused on identifying germline CNVs associated with breast cancer susceptibility and prognosis.

Since germline structural variations and their coverage on the genome is higher than SNPs, we reasoned that CNVs are suitable candidates to explore for their associations with prognosis. Germline CNVs have been identified as prognostic markers for several cancer types including prostate cancer^54^, ovarian cancer^25^ and colorectal cancer^55^. Our group showed that germline Copy Neutral Loss of Heterozygosity (CN-LOH), a class of CNVs, are associated with recurrence free survival in breast cancer^56^.

Our aim was to conduct GWAS to identify common germline CNVs associated with breast cancer risk and assess if subsets of the risk associated CNVs are also associated with prognosis. Earlier studies on CNV association in familial breast cancer were restricted to identifying disease risk variants but not prognosis^18–20^. Specifically, we conducted CNV-GWAS, firstly focusing on identifying common CNVs overlapping with protein coding genes for association with breast cancer risk, secondly investigating the prognostic significance of the risk associated CNVs and thirdly correlating breast cancer risk associated CNVs with breast tumor tissue specific gene expression. We have identified several common CNVs associated with breast cancer and determined that subsets of these CNVs are associated with both disease risk and prognosis. These findings highlight the importance of pursuing common germline CNVs to address the knowledge gap in the literature.

## Results

### A) CNV-GWAS: Identification of breast cancer associated CNVs in coding regions

**Figure 1:**
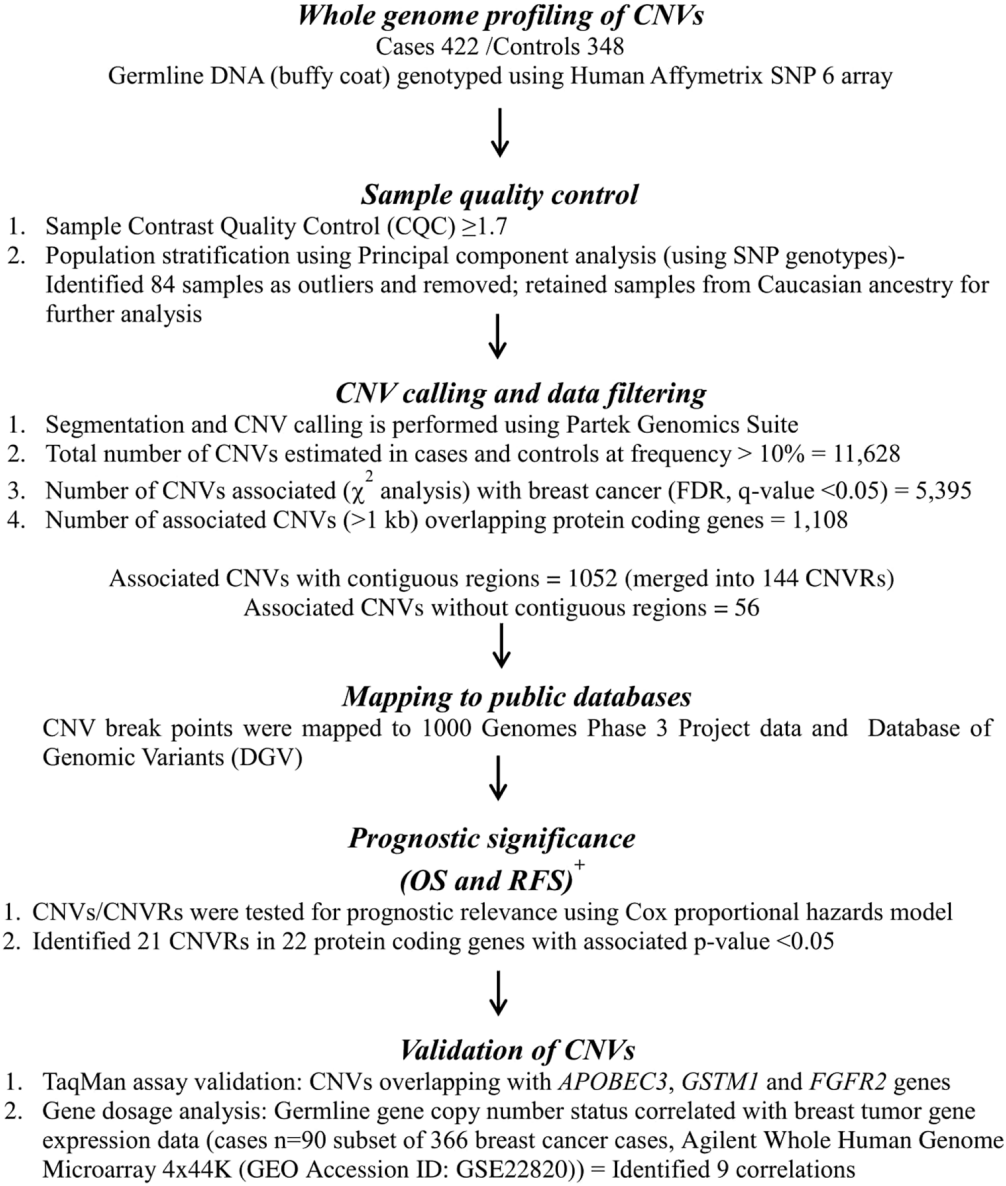
Study Overview. The figure outlines the study design with brief description of methods and data filters. Summary of key result of each analysis indicating the number of CNVs at various stages of analysis. OS, overall survival; RFS, recurrence free survival. + Time to event analysis based on cases (n = 366).

We identified 11628 CNVs in autosomes in an analysis that was restricted to common variants at frequency > 10% in the study samples (see Fig. 1 for study design). CNV frequencies compared between cases and controls (2 × 3 chi-square test) resulted in identification of 5395 CNVs which were statistically significantly associated with breast cancer at q-values < 0.05. We only considered CNVs with size more than 1 kb for further analysis to increase confidence in CNV segments estimated by the algorithm. Although we identified CNVs in both protein coding and non-coding genes, those overlapping protein-coding genes have higher potential to contribute to phenotypic variation^44^ and we therefore focussed on identification of CNVs overlapping with protein coding genes. CNVs were annotated for protein coding genes using RefSeq (GRCh37/ Human genome, Hg19 build) gene annotations. Of the 5395 CNVs that were significantly associated (q < 0.05) with breast cancer, 1108 CNVs were mapped to 258 protein coding genes. We merged multiple contiguous CNVs from the set of 1108 into a single Copy Number Variable Region (CNVR) and interrogation of the overlapping genes for association with breast cancer yielded 200 altogether (144 CNVRs and 56 CNVs). The size ranges of the CNVRs and CNVs were 1.1–237 kb and 1.1–9 Mb, respectively. The list of all associated CNVs/CNVRs is given in Supplementary Table S1 and the list of the top CNVRs/CNVs (with q-values < 10^−5^) is given in Table 1.

**Table 1:** Top associated germ line CNVs/CNVRs associated with breast cancer risk.

CNV region	Cytoband	Size (bp)	Total CNV /CNVR Frequency in cohort	Average Frequency of CNV		q-value	Overlapping gene	Mapping
				Cases (Gain/Loss)	Controls (Gain/Loss)			
Chr5-69784291-70254895	5q13.2	470605	44	31 (13/18)	59 (3/56)	1.46 × 10^−21^	*SMN2*, *ERF1A*, *GUSBP9*, *SERF1B*, *SMN1*, *SMA5*, *GUSBP3*,	1000 g, DGV
Chr5-70254905-70328368	5q13.2	73469	31	26 (11/15)	37 (7/30)	3 × 10^−02^ to 1.76 × 10^−13^	*NAIP*	1000 g. DGV
Chr21-40184963-40190820	21q22.2	2792	15	7 (3/4)	24 (0/24)	1.58 × 10^−10^ to 4.3 × 10^−12^	*ETS2*	—
Chr9-40784158-40800446	9p13.1	60428	19	12 (5/7)	28 (3/25)	1.09 × 10^−11^ to 5.23 × 10^−12^	*ZNF658*	DGV
Chr8-7827144-7831849	8p23.1	4707	24	15 (7/8)	33 (4/29)	1.02 × 10^−09^ to 1.65 × 10^−09^	*FAM66E*, *USP17L8*	DGV
Chr9-67899911-68067313	9q13	167404	18	8 (2/6)	29 (4/25)	1.86 × 10^−08^ to 1.52 × 10^−09^	*ANKRD20A1*, *ANKRD20A3*	DGV
Chr1-248683401-248687808	1q44	4409	29	23 (8/15)	35 (1/34)	2.38 × 10^−08^ to 6.47 × 10^−09^	*OR2G6*	DGV
Chr11-55418110-55421252	11q11	3143	85	94 (49/45)	76 (32/44)	1.21 × 10^−08^	*OR4S2*	1000 g, DGV
Chr8-93005629-93015066	8q21.3	9444	11	5 (2/3)	18 (0/18)	7.69 × 10^−08^ to 5.94 × 10^−09^	*RUNX1T1*	—
Chr6-34516636-34517772	6p21.31	1143	11	17 (13/4)	6 (0/6)	1.34 × 10^−07^ to 1.02 × 10^−08^	*SPDEF*	DGV
Chr11-55403771-55407672	11q11	3902	85	93 (49/44)	77 (33/44)	4.18 × 10^−08^	*OR4P4*	1000 g, DGV
Chr1-149548719-149563724	1q21.2	15005	30	26 (10/16)	35 (2/33)	6.61 × 10^−08^	*PPIAL4A*, *PPIAL4C*	1000 g, DGV
Chr10-123346484-123348045	10q26.13	1569	11	7 (3/4)	15 (0/15)	6.04 × 10^−07^ to 1.05 × 10^−07^	*FGFR2*	—
Chr16-10788745-10790882	16p13.13	2137	10	7 (4/3)	14 (0/14)	4.24 × 10^−07^	*TEKT5*	1000 g, DGV
Chr1-356492-380356	1p36.33	23865	21	16 (8/8)	28 (4/24)	5.62 × 10^−07^	*OR4F16*, *OR4F29*, *OR4F3*	1000 g, DGV
Chr9-67789400-67808579	9q13	19180	19	10 (2/8)	28 (3/25)	7.98 × 10^−07^	*FAM27B*	1000 g, DGV
Chr4-144288613-144293270	4q31.21	4667	18	11 (5/6)	26 (2/24)	1.5 × 10^−05^ to 2.4 × 10^−11^	*GAB1*	DGV
Chr4-69505724-69536970	4q13.2	31250	32	29 (12/17)	35 (5/30)	1.29 × 10^−03^ to 1.10 × 10^−06^	*UGT2B15*	1000 g, DGV
Chr11-55430518-55436423	11q11	5907	81	87 (46/41)	73 (30/43)	1.68 × 10^−05^ to 2.79 × 10^−08^	*OR4C6*	DGV
Chr9-67753281-67808579	9q13	55300	19	11 (2/9)	28 (3/25)	1.46 × 10^−06^ to 7.87 × 10^−07^	*FAM27E3*,	1000 g, DGV
Chr13-67509369-67513167	13q21.32	3811	11	7 (3/4)	14 (1/14)	1.24 × 10^−03^ to 2.07 × 10^−06^	*PCDH9*	DGV
Chr7-75044860-75062133	7q11.23	17277	12	7 (3/4)	17 (0/17)	2.09 × 10^−06^ to 1.76 × 10^−07^	*NSUN5P1*, *POM121C*	DGV
Chr17-20346165-20366887	17p11.2	20725	11	7 (3/4)	15 (0/15)	2.08 × 10^−06^ to 6.78 × 10^−07^	*LGALS9B*	1000 g, DGV
Chr4-55106768-55120708	4q12	13940	17	15 (6/9)	19 (0/19)	5.21 × 10^−03^ to 6.14 × 10^−08^	*PDGFRA*	—
Chr13-48968806-48977635	13q14.2	8835	11	7 (3/4)	17 (0/17)	1.53 × 10^−06^ to 6.19 × 10^−07^	*RB1*	1000 g
Chr3-127422064-127423993	3q21.3	1931	10	6 (2/4)	15 (0/15)	6.29 × 10^−06^ to 4.01 × 10^−06^	*MGLL*	1000 g, DGV
Chr5-180425664-180437832	5q35.3	12170	19	19 (9/10)	18 (1/17)	4.71 × 10^−05^ to 2.62 × 10^−05^	*BTNL3*	1000 g, DGV
Chr1-152572873-152574332	1q21.3	2728	75	83 (40/43)	67 (24/43)	4.71 × 10^−05^ to 2.64 × 10^−05^	*LCE3C*	1000 g, DGV
Chr22-39363651-39371629	22q13.1	1119	19	21 (3/18)	17 (3/14)	3.65 × 10^−02^ to 2.73 × 10^−02^	*APOBEC3A_B*	1000 g, DGV

List of CNVs/CNVRs identified in the CNV-GWAS that were associated (q-value < 5 × 10^−5^) with breast cancer. For CNVRs, we presented the range of q-values from the CNVs identified (Supplementary 1 Table S1). The last row shows the CNVR from *APOBEC3A_B* (fusion gene) reported in the literature^47^ and its association with breast cancer risk in the current study as an independent validation of findings.

### (i) Mapping of CNVs to publicly available structural variation databases

Different genomic segmentation algorithms have their strengths and limitations^57^; the CNV break points called by different algorithms may or may not overlap and some algorithms tend to overcall CNVs^57^. Therefore, it was important to ascertain that the called CNVs were reliable by independent methods, and CNVs were mapped to the DGV and 1000 Genomes Project phase 3 data to assess concordances for the CNVs identified in this study. Ninety percent of CNVs associated with breast cancer mapped to the DGV, and while this is a common approach, this database has limitations. DGV curation is ongoing; its datasets are generated on diverse microarray platforms and by diverse CNV calling algorithms^57^. We, therefore, considered a second method using higher resolution structural variation data available in the public domain from the 1000 Genomes Project (Phase 3). We mapped 76% of the 200 CNVRs/CNVs to the 1000 Genomes Project data and most of these (94%) also had hits in DGV, giving confidence in the CNV calling methods utilised in this study.

### B) CNVRs associated with breast cancer prognosis

Since SNPs associated with breast cancer risk are poor prognosticators^52^, we investigated if the CNVs associated with breast cancer risk would have prognostic significance. We tested the 200 CNVRs/CNVs that showed association with breast cancer risk for prognostic significance using the Cox proportional hazards model. We compared the hazard function among the cases with diploid gene copy versus copy gain or loss. The identified prognostic CNVRs for Overall Survival (OS) and Recurrence Free Survival (RFS) are summarized in Tables 2 and 3. We identified 21 CNVRs overlapping 22 genes that showed associations with both breast cancer risk and prognosis.

**Table 2:** CNVRs associated with breast cancer risk and OS.

CNVR region	Gene name	CNVR Size (kb)	Copy number status	P-value	Hazards Ratio [95% CI]
chr19:36846012-36847567*	*ZFP14*	1.55	gain	4.78 × 10^−3^	2.38 [1.3-4.36]
chr1:65393459-65410228*	*JAK1*	16.77	gain	1.07 × 10^−2^	3.24 [1.31-8.01]
chr1:110225034-110226615	*GSTM2*	1.58	gain	1.30 × 10^−2^	1.81 [1.13-2.89]
chr17:80646036-80647251	*RAB40B*	1.21	gain	1.60 × 10^−2^	2.57 [1.19-5.52]
chr6:32487136-32497161	*HLA-DRB5*, *HLA-DRB6*	10.02	gain	2.25 × 10^−2^	0.59 [0.38-0.93]
chr8:72213838-72215337	*EYA1*	1.49	gain	3.09 × 10^−2^	1.59 [1.04–2.43]
chr6:161032642-161068568*	*LPA*	35.92	gain	3.13 × 10^−2^	0.37 [0.15–0.91]
chr3:50951343-50960775	*DOCK3*	9.43	gain	3.18 × 10^−2^	2.20 [1.07–4.52]
chr12:99796328-99797863	*ANKS1B*	1.53	gain	3.35 × 10^−2^	1.94 [1.05–3.57]
chr12:2254285-2256046	*CACNA1C*	1.76	gain	3.49 × 10^−2^	0.48 [0.24–0.95]
chr4:55111660–55120708*	*PDGFRA*	9.05	loss	6.58 × 10^−3^	0.35 [0.16–0.74]
chr16:515664-536683	*RAB11FIP3*	21.02	loss	1.66 × 10^−2^	0.43 [0.22-0.86]
chr21:11053457-11069332	*BAGE*	15.87	loss	2.01 × 10^−2^	0.40 [0.19–0.87]
chr8:14284477-14288732	*SGCZ*	4.25	loss	2.41 × 10^−2^	0.27 [0.08–0.84]
chr7:75044860-75054268	*POM121c*	9.41	loss	4.77 × 10^−2^	0.20 [0.06–0.98]

List of CNVRs associated with both risk and overall survival identified using Cox proportional hazard model. Only the associated copy number status (either loss or gain) compared with diploid is indicated in the table. The CNVR region marked with “*” indicate common CNVRs between OS and RFS. Abbreviation: CI – Confidence Interval.

**Table 3:** CNVRs associated with breast cancer risk and RFS.

CNVR region	Gene name	CNVR Size (kb)	CNV type	Cox P-value	Hazards Ratio [95% CI]
chr19:36846012–36847567*	*ZFP14*	1.55	Gain	3.82 × 10^−4^	2.89 [1.61–5.19]
chr4:186629984-186634169	*SORBS2* ^+^	4.18	Gain	1.35 × 10^−2^	3.54 [1.3–9.64]
chr1:152572873-152574332	*LCE3C*	1.46	Gain	1.94 × 10^−2^	1.75 [1.1–2.81]
chr1:248787969-248794876	*OR2T11*	6.91	Gain	2.64 × 10^−2^	2.09 [1.09–4]
chr3:195456468-195461506	*MUC20*	5.04	Gain	3.46 × 10^−2^	0.62 [0.39–0.97]
chr1:65393459-65410228*	*JAK1*	16.77	Gain	3.47 × 10^−2^	2.6 [1.07–6.47]
chr6:161032642-161068568*	*LPA*	35.92	Gain	5.08 × 10^−3^	0.31 [0.13–0.70]
chr17:20346165-20366887	*LGALS9B*	20.72	Gain	3.52 × 10^−2^	2.27 [1.06–4.87]
chr4:55111660-55120708*	*PDGFRA*	9.05	Loss	7.92 × 10^−3^	0.42 [0.22–0.8]
chr6:53931117-53933601	*MLIP*	2.48	Loss	2.53 × 10^−2^	0.62 [0.4–0.94]
chr4:186629984-186634169	*SORBS2* ^***+***^	4.18	Loss	3.65 × 10^−2^	1.93 [1.04–3.58]

List of CNVRs associated with both risk and RFS identified using Cox proportional hazard model. Only the associated copy number status (either loss or gain) compared with diploid is indicated in the table. The CNVR region marked with “*” indicate common CNVRs between OS and RFS “** + **” Indicates that gene that has both gain and loss associated with recurrence free survival when compared to diploid. Abbreviation: CI – Confidence Interval.

### (i) Germline CNVRs and OS in Breast cancer

**Figure 2:**
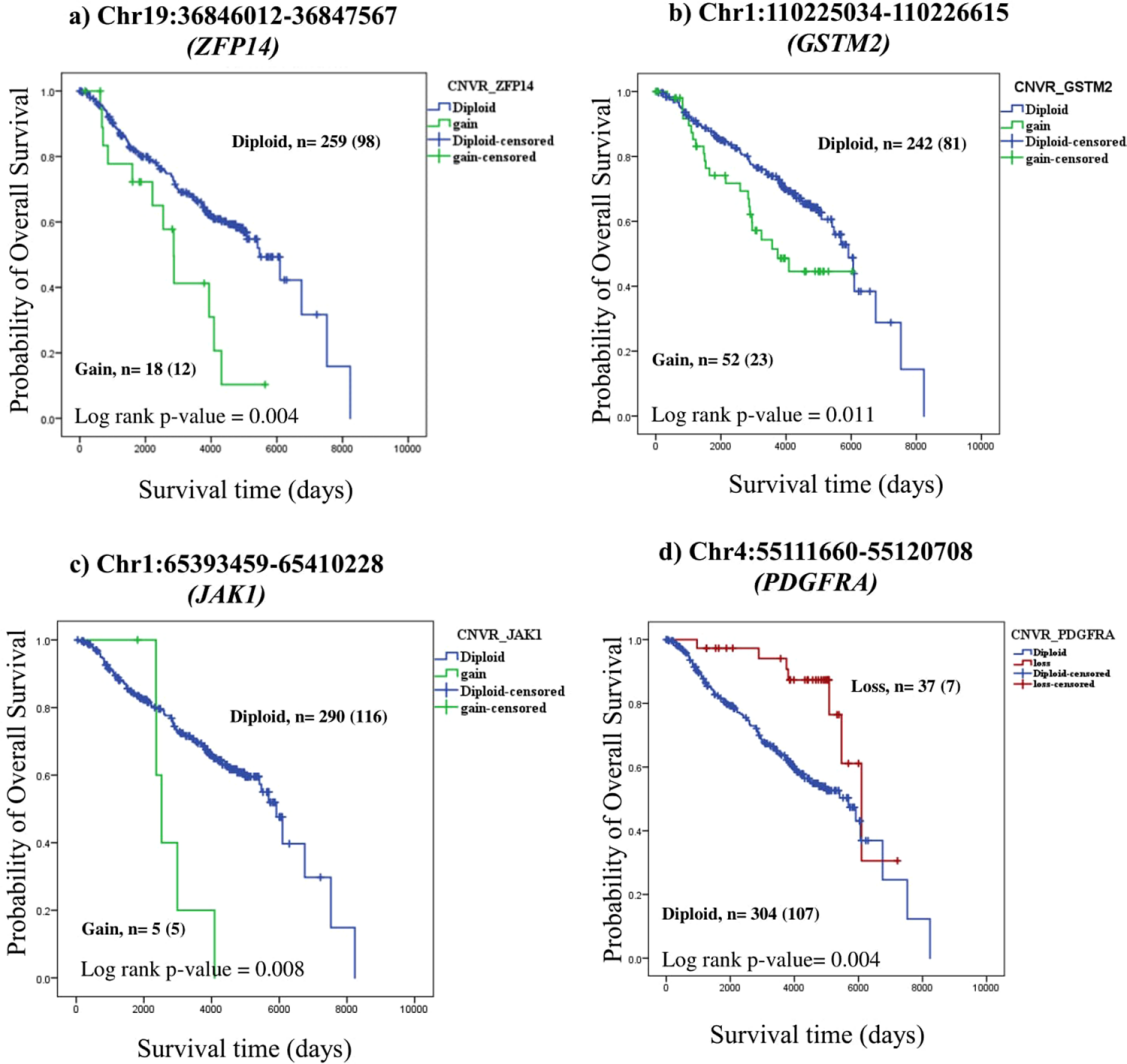
Kaplan Meier plots for CNVRs associated with Overall Survival. KM plots were constructed based on the copy number status of each gene to determine the difference in overall survival (OS) between cases with genes harbouring copy number variation (gain/loss) versus diploid status. Blue indicates Diploid copy number; Green indicates Copy number gain; Red indicates Copy number loss. “ + ” indicates the censored events. The number of cases, n, in the analysis is indicated and the number of events in the study for each survival curve is indicated in parenthesis. Log rank p-value for significance between the curves is indicated at the bottom of each panel within the figure.

We identified 15 CNVRs (with 16 overlapping genes) associated with breast cancer risk and OS (Table 2). Among these, 11 CNVRs overlapped with 12 genes (*GSTM2*, *RAB40B*, *HLA_DRB5*, *HLA_DRB6*, *EYA1*, *DOCK3*, *ANKS1B*, *CACNA1C*, *RAB11FIP3*, *BAGE*, *SGCZ*, *POM121c*) and were specifically associated with breast cancer risk and OS. The remaining four CNVRs overlapped with genes *ZFP14*, *JAK1*, *LPA*, *PDGFRA* and were also associated with RFS in breast cancer. The P-values for the identified 15 CNVRs were in the range of 4.77 × 10^−2^ to 4.78 × 10^−3^. Both gains and losses contributed to prognostic significance. Copy gains showed both risk elevating and protective effects whereas copy losses showed only protective effects. The Kaplan-Meier (KM) survival plot for the top associated CNVR with OS is shown in Fig. 2. Copy number gains in the genes *ZFP14*, *GSTM2* and *JAK1* were shown to be associated with poor OS in the univariate Cox analysis (Fig. 2a-c). P-values and HRs estimated for these genes were as follows: *ZFP14* (P-value = 4.78 × 10^−3^ and HR 2.38), *GSTM2* (P-value = 1.30 × 10^−2^ and HR 1.81) and *JAK1* (P-value = 1.07 × 10^−2^ and HR 3.24). KM plots describing the survival differences and estimated log rank p-values are shown in Fig. 2a–d. The estimated survival differences (log rank p-values) for cases with copy gains compared to cases with diploid copies of the genes *ZFP14*, *GSTM2*, and *JAK1* were 0.004, 0.11 and 0.008 respectively. Copy number loss of *PDGFRA* was associated with OS (P-value 6.58 × 10^−3^ and HR 0.35) and cases with copy loss had better survival outcomes compared with cases with diploid copies, the log rank p-value estimated for the difference in survival was 4 × 10^−3^.

### (ii) Germline CNVRs and RFS in Breast cancer

**Figure 3:**
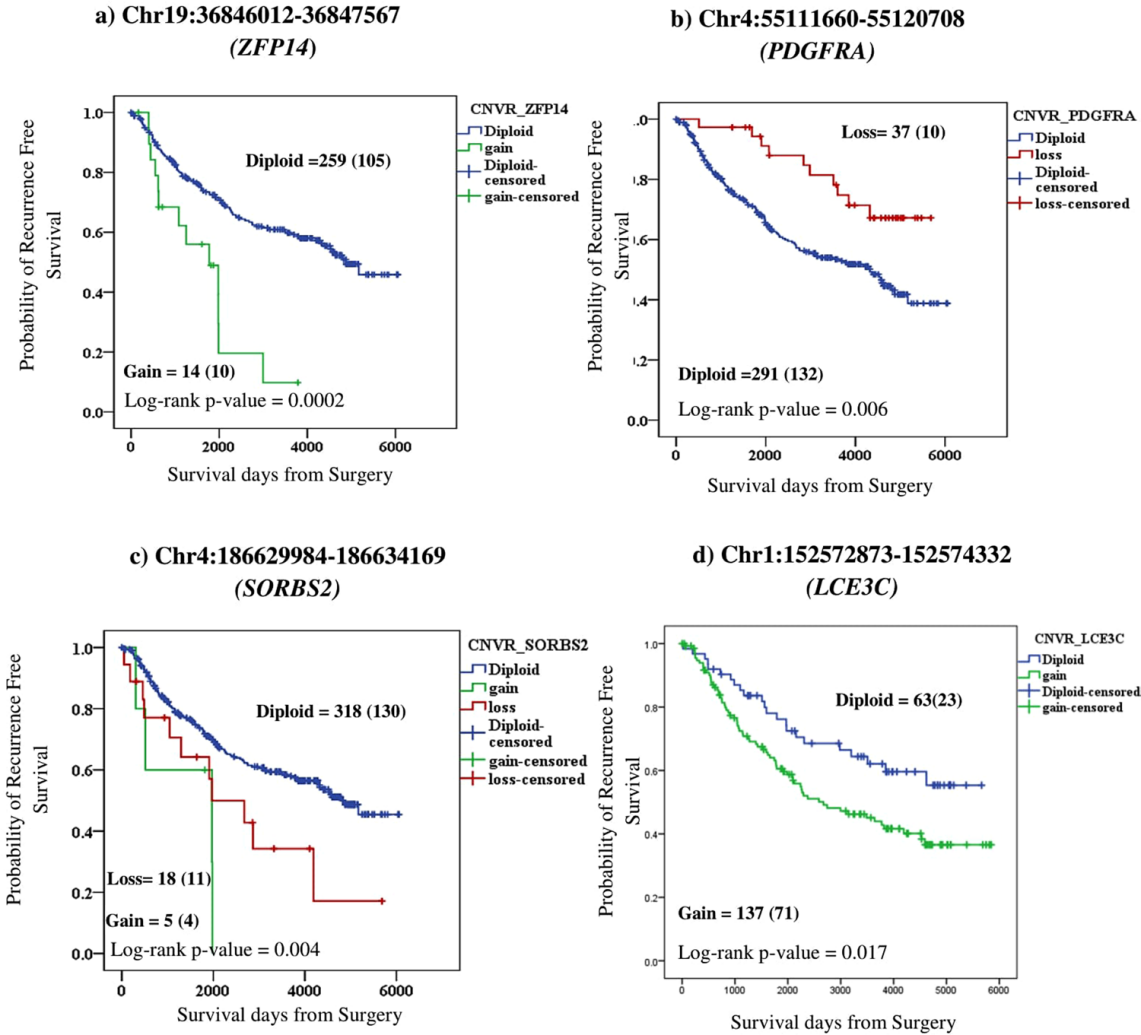
Kaplan Meier plots for CNVRs associated with Recurrence Free Survival. KM plots were constructed based on the copy number status of each gene to determine the difference in recurrence free survival (RFS) between cases with genes harbouring copy number variation (gain/loss) versus diploid status. Blue indicates Diploid copy number; Green indicates Copy number gain; Red indicates Copy number loss. “ + ” indicates the censored events. Number of cases, n in the analysis is indicated and the number of events in the study for each survival curve is indicated in parenthesis. Log rank p-value for significance between the curves is indicated at the bottom of each panel within the figure.

We identified a total of ten CNVRs associated with breast cancer risk and RFS (Table 3). Among the ten CNVRs, six CNVRs overlapped with the genes (*SORBS2*, *LCE3C*, *MLIP*, *OR2T11*, *MUC20*, *LGALS*) that were specifically associated with RFS; and four CNVRs (*ZFP14*, *JAK1*, *LPA*, *PDGFRA)* were also associated with OS. The associated CNVRs had P-values in the range of 3.65 × 10^−2^ to 3.82 × 10^−4^. Both copy gains and losses were associated with elevated risk or protective effects. The KM plots for the top associated CNVRs with RFS are illustrated in Fig. 3. We observed that copy gains in *ZFP14* and *LEC3C* were associated with poor RFS with P-values 3.82 × 10^−4^ and 1.94 × 10^−2^ and HRs 2.89 and 1.75, respectively. The log rank p-value estimated from KM plots (Fig. 3a,d) for the genes ZFP14 and *LEC3C* were 2.0 × 10^−4^ and 1.7 × 10^−2^, respectively. In *PDGRA* gene copy loss associated with RFS and cases with copy loss had better survival outcomes compared with diploid copy status (RFS, P-value 7.92 × 10^−3^ and HR 0.42). The log rank p-value estimated was 6 × 10^−3^ based on KM plot (Fig. 3b). A similar trend was observed for OS as well. Another interesting CNVR was in the SORBS2 gene in which both copy gain and loss were associated with poor RFS. For copy gain, the P-value was 1.35 × 10^−2^ and HR was 3.54; for copy loss, the P-value was 3.65 × 10^−2^, and the HR was 1.93. The log rank p-value for the difference in the copy gain/loss versus diploid copy status was 4 × 10^−3^ **(**Fig. 3c **)**.

We observed that copy number deletion in *APOBEC3A_B* was not associated with either RFS and OS in breast cancer, which agrees with published findings^58^.

## Validation of associated CNVs

### Cross platform validation of CNVs using the TaqMan Assay

**Figure 4:**
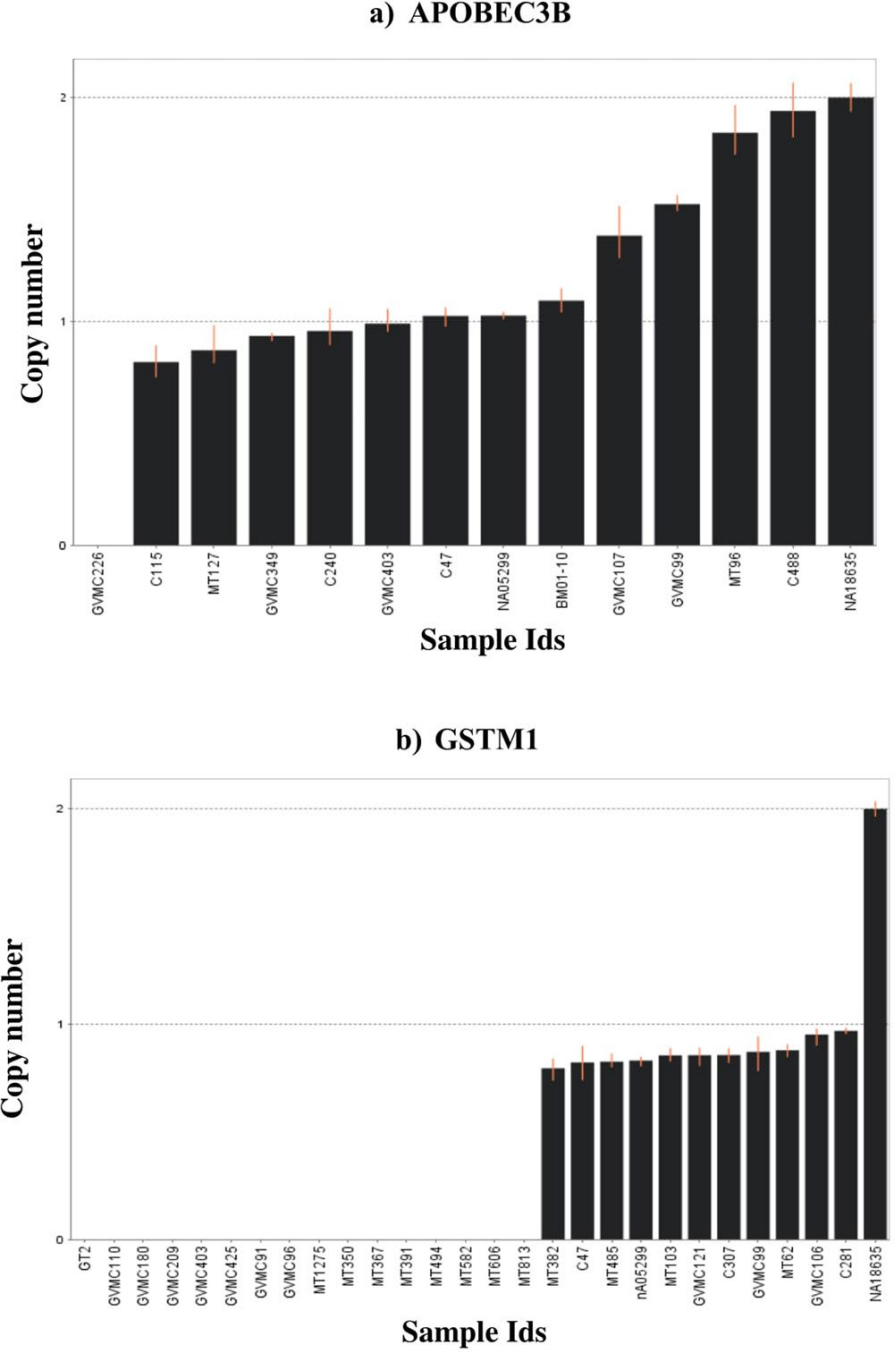
Copy number status estimated in study samples using TaqMan Assay. Copy number status of genes *APOBEC3B* (**a**) and *GSTM1* (**b**) are represented for each sample. The Human *RNAase P* was used as internal normalization and the Coriell sample NA18635, which is diploid for both genes, were also used in copy number estimation.

Breast cancer associated CNVs overlapping with the genes *APOBEC3B*, *GSTM1 and FGFR2* were validated using the TaqMan assay. For APOBEC3B, 13 samples were tested **(**Fig. 4a **)**: one sample (healthy control) had two copy deletions, ten samples had one copy deletion (4 healthy controls and 6 breast cancer cases) and two samples (breast cancer cases) had diploid copy numbers. For *GSTM1*, we identified 16 samples (7 controls, 9 cases) with two copy deletions and 11 samples (3 controls and 8 cases) with one copy deletion (Fig. 4b **)**. Both *APOBEC3* and *GSTM1* quantifications by the TaqMan assays showed excellent agreement with the predicted copy status from PGS (this study) and the 1000 genomes data.

CNVs identified in *FGFR2* predominantly showed copy deletions as inferred by PGS; the same CNVs, when mapped to the 1000 genomes data, showed diploid status. We tested 29 samples (19 controls and 10 cases) by the TaqMan assay to verify copy status; all samples showed diploid status. To ensure the quality of the assay design, we used the Coriell DNA sample (NA05299) that had one copy deletion in *FGFR2* as a positive control for *FGFR2* deletion thereby demonstrating that the technical aspects of the TaqMan assay did not contribute to disagreement in the copy deletions noted (data not shown). A targeted re-sequencing of this region is needed to confirm these findings.

### Detailed characteristics of the validated CNVs

(a) *APOBEC3A_B* loci: A deletion of *APOBEC3A_B* was previously reported to be associated with breast cancer risk in Chinese^47^, European^48^ and Iranian^59^ populations. In this study, we also identified CNVs showing a deletion in the *APOBEC3B* gene and associated with breast cancer risk (Table 1). We validated the deletion in our cohort using the TaqMan assay as an independent genotyping platform. A single copy deletion of *APOBEC3A_B* was observed at frequencies of 14% among controls and 18% of cases (Caucasian ancestry), which is comparable with results of previous reports^48^. This is the second such study based on a Caucasian population to independently validate a common CNV and its association with breast cancer.

(b) *GSTM1*: Although the role of germline CNVs in the *GSTM* family of genes, which are involved in xenobiotic detoxification and drug metabolism pathways, is well documented in other cancer types^60^, their role in breast cancer is not clear. We identified CNVs (both gains and losses) in *GSTM1* and *GSTM2* and their frequencies in the total cohort were 78% and 27% in the Caucasian population, respectively (Supplementary Table S1). The relative frequencies of deletions in *GSTM1* (Cases, 40%; Controls, 31%) and *GSTM2* (Cases, 15%; Controls, 8%). CNVs were higher among the cases compared to the controls. The CNVs identified in *GSTM* loci were also observed in 1000 Genomes Project data as a copy variable region.

### Correlation of germline CNV copy status of protein coding genes with gene expression in breast tumors

**Figure 5:**
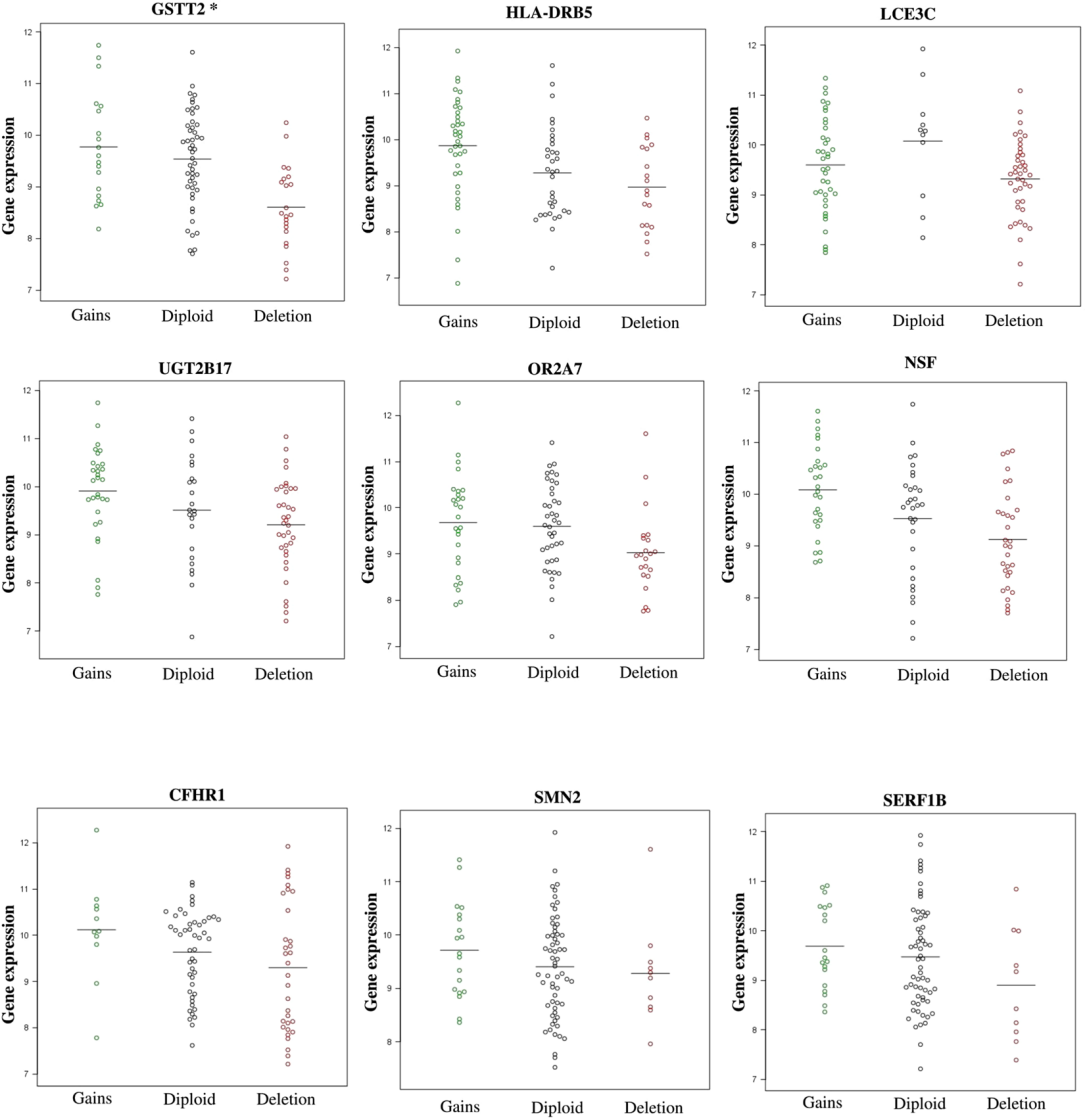
Association of germline copy number status and gene expression in breast tumor tissue. Germline copy number status of individual genes was plotted against gene expression in breast tumors from matched samples. The colours indicated in green, grey and red represent gain, diploid and deletion, respectively.

One of the mechanisms by which germline CNVs may bring about phenotypic effects is gene dosage, and in this context “functionality” refers to underlying gene expression changes in breast tumor tissues rather than specific changes in cellular morphology or proliferation rates. To identify gene dosage effects due to germline CNVs, we looked for correlations between gene expression profiles derived from breast tumor biopsy samples (n = 90) and the germline CNV data available from the same cases. We expected only a subset of genes to be expressed in a tissue specific manner and our observations support this premise. The expression of nine genes correlated with corresponding germline CNVs with correlation coefficients in the range 0.2 to 0.39 (Supplementary Table S2). Seven of the nine genes also were statistically significant at p < 0.05 and two showed trends of association (p < 0.1). The association of gene expression as a function of the germline copy number status is illustrated in Fig. 5. Mean expression levels among cases with copy number deletions were consistently less among cases compared to  diploid copy number or amplification. The correlated genes identified here are well known to harbour germline copy number variations^61–63^, and the association of CNVs in these genes with breast cancer risk and the altered expression of these genes in breast tumor tissues is noteworthy.

In addition to the linear correlation of gene expression with CNVs, we also tested if the genes overlapping in the prognostic CNVs (n = 22) were also associated with RFS and OS. Eighteen of the 22 genes overlapping in the CNVRs also showed expression in breast tumor tissues. Of these, expression of five genes *(GSTM2*, *SGCZ*, *HLA_DRB5*, *ZFP14*, *LCE3C*) showed association with prognosis (Supplementary Table S3).

## Discussion

In this study, we sought to identify germline CNVs that predispose to both breast cancer susceptibility and prognosis. Using 686 samples for copy number analysis, we identified 200 CNVs/CNVRs (frequencies > 10%) that overlapped with protein coding genes at q-values < 0.05. We compared the identified CNVs/CNVRs break points to the structural variation data available from the 1000 Genomes Project to ascertain CNV calls, an approach that was unique to our study. Another novel aspect was the assessment of prognostic relevance of breast cancer susceptibility CNVs. We demonstrated that some CNVs were only associated with disease risk whereas some were associated with both disease risk and prognosis. Our findings are in contrast to SNP based association studies in which susceptibility SNPs from GWAS did not show prognostic relevance, with one exception, the SNP rs13281615^64^ on chromosome 8q24.21 locus which we and others showed as associated with both OS and RFS in breast cancer^51^. Further, independent SNP based GWAS were not successful in identifying variants associated with breast cancer prognosis^52^. CNVs cover 10% of the genome based on nucleotide coverage and our study rationale assumed that CNVs overlapping with coding genes (deletions or gains) influence phenotypes.

Of relevance was the replication in our study of the *APOBEC3A_B* gene deletion (Chr22-39363651-39364770), which was originally reported in Chinese populations as a breast cancer susceptibility CNV in sporadic cases^47^. Subsequently the same was replicated in European^48^ and Iranian populations^59^. There were both gains and losses at this locus in this study; frequencies of gains were the same in both cases and controls (at 3%) whereas the above published studies reported only copy loss. The copy number deletion is the risk allele and the frequencies were 18% and 14%, respectively, in cases and controls (this study). These were in agreement with reported studies^65^ in Caucasian populations (Table 1). *APOBEC3B* gene was not shown to be associated with prognosis (OS)^58^, which we confirmed in this study.

We have identified a CNV (Chr1:110230244-110233070) showing association with breast cancer and harbouring the *GSTM1* gene. Earlier candidate gene studies identified SNPs in *GSTM1* to be associated with breast cancer risk^66^. We report a common CNV approximately 3 kb in size in a locus encompassing *GSTM1* associated with breast cancer risk. The 1000 genome annotation indicates that a CNV in this genomic locus spans about 20 kb in size and encompasses the entire gene. The CNV encompassing *GSTM1* showed both gains and losses at high frequencies in cases and controls (Supplementary Table S1). The frequencies were approximately the same for gains in cases and controls (43% vs. 42%). However, deletion frequencies differed between cases and controls (40% vs. 31%), with cases showing higher frequencies. Although a germline CNV overlapping *GSTM1* was shown to be associated with prognosis in prostate and bladder cancers^60^, this CNV was not associated with prognosis in this study. SNP based studies in the *GSTM1* gene SNPs associated with breast cancer risk but not with prognosis^67,68^. We validated both *APOBEC3 and GSTM1* CNV deletions using the TaqMan assays. Interestingly, the representative genes *(APOBEC3B and GSTM1)* validated by the TaqMan assays were also identified as copy variable genes by the 1000 genomes project.

The characteristics and putative biological roles for representative genes associated with breast cancer susceptibility and/or prognosis are summarized here:

(i) *PDGFRA*, Platelet-Derived Growth Factor Receptor Alpha is a tyrosine kinase receptor that is overexpressed in malignancies including the breast. We observed a CNV in *PDGFRA* is not only associated with BC risk and but a copy loss in this gene is conferring protective effect for RFS and OS. A higher frequency of copy gain was seen in cases (~6%) compared to 0% frequency among controls. However, frequency of deletion observed in controls was higher (19%) compared to cases (9%). Overexpression of *PDGFRA* is also known to play a role in tumorigenesis and its amplification or genetic alteration is believed to activate the *PDGFRA* mediated signalling pathway^69^.
(ii) *LPA* (Lysophosphatidic acid), a lipid biomolecule that functions as a growth factor mediating cell proliferation, migration and progression, processes that are central to tumorigenesis^70,71^. Both CNV and gene expression profiles of LPA are associated with both susceptibility and prognosis. Copy number gain was associated with protective effect for OS and RFS.
(iii) A germline CNV in *ZFP14* (Zinc Finger protein) was associated with risk and prognosis in our analysis. CNV in *ZFP14* is associated with prostate cancer^23^, in which a deletion is protective for prostate cancer risk. We observed a copy gains among the cases that was associated with poor prognosis. Somatic copy number aberration is also observed in *ZFP14* gene in breast tumors^72,73^.

The CNV association studies in breast cancer reported thus far have focused on cases that are BRCA positive or with family history with or without BRCA mutations^18^ and with limited sample sizes (n = 30–60). These studies identified rare CNVs (frequency < 1% in total cohort). Recently a CNV-GWAS study was conducted using cases with early onset of breast cancer (age < 40 Years; 200 cases and 293 controls) and genotyping was performed using Illumina Human610-Quad BeadChip^15^ and CNV calls were inferred based on SNP probe intensities. Our study utilized cases that were diagnosed with invasive breast cancer with late age at onset of the disease (>40 Years; 422 cases and 348 controls) and focused on common CNVs. We used Affymetrix SNP 6 arrays and CNV calls were based both on SNP and CNV probes. Because SNP density is lower in CNV dense regions, our study benefitted from using the Affymetrix arrays. Most existing studies on CNV associations with breast cancer have relied on SNP probes, and CNV calling algorithms are also diverse. Hence potential overlap of the genes identified in our study with those previously described are likely to be highly restrictive. Our use of both CNV and SNP probes to infer copy status may have contributed to higher numbers of CNVs associated with breast cancer. As with any GWAS study, Stage-1 study identifies several variants associated with the phenotype, and our data conforms with the GWAS literature. However, we addressed multiple hypothesis testing by implementing q-value (<0.05) thresholds. In addition, we also mapped the associated CNVs with breast cancer to 1000 Genomes Project database and confirmed that a majority of CNVs identified were indeed common CNVs. We have replicated CNVs (n = 5) from the familial breast cancer study, including CNVs in genes *ANKS1B* ^19^, *OR4C11*, *OR4P4*, *UGT2B17*, *OR4C6*, *OR4S2* ^15^. Even though previous studies have ascribed these CNV overlapping genes to early onset of breast cancer, independent replication of these findings in late age at onset of breast cancer (this study) suggests that some CNVs may be common and emphasizes the more general role these genes play in the aetiology of breast cancer.

The breast cancer risk associated CNVs (Table 1) that mapped to 1000 genomes *(NME7*, *RB1*, *UGT2B15*, *BTNL3*, *RBL1*, *LGALS9B*, *MGLL*, *GSTM1*, *and PML*) were also captured in a recent breast tumor tissue (somatic) profiling study, confirming that the identified genes are primarily in copy number variable regions^73^.

We tested the 200 CNVRs overlapping protein coding genes for their associations with breast cancer RFS and OS using the Cox proportional hazard model. The cases in our study have well annotated clinical data and long years of follow up, and we compared the survival benefit of cases based on the germline copy number status (gain or loss) against diploid copy for a given CNVR. We identified CNVRs to be associated with RFS and/or OS among the cases. Genes within the four CNVRs (*i*.*e*., *ZFP14*, *JAK1*, *LPA*, *PDGFRA*) were associated with both RFS and OS; these genes are also known to harbour somatic copy number aberrations in breast tumors^72–74^.

It is critical to demonstrate the functionality of genes overlapping with CNVs. We therefore examined their dosage sensitivities and identified nine genes whose expression is breast tissue specific. The dot plots (Fig. 5) clearly indicate the differences in expression levels between deletion versus diploid genes. The well-known germline CNV harbouring genes, *GSTT1*, *UGT2B17*, are involved in detoxification, steroid and drug metabolism pathways. and their dosage sensitivities are well studied^67,75,76^. These genes are also associated with breast cancer risk and demonstrating dosage sensitivity at the tissue level will contribute to an understanding of the mechanistic basis for disease aetiology. Even though GST family of genes showed associations at the CNV level, their correlation with gene expression was not significant due to the unequal distribution of samples across different copy number states and the limited sample size of 90. A larger sample size with gene expression and germline CNV profiles will allow us to detect correlations between CNVs and gene expression.

## Conclusion

Our study restricted the analysis to CNVs overlapping with protein coding regions, the preferred approach in most CNV based association studies reported in the literature^44,47^. Although intergenic CNVs in non-coding regions also merits attention, access to matched data sets (germline CNVs and gene expression data) is needed and these are to be addressed in future studies. Such data mining approaches have shown promising leads in disease settings other than breast cancer^77,78^. In this study, we identified CNVs associated with breast cancer phenotypes, vis-à-vis, heritable determinants for disease susceptibility and prognosis and predict that our results also apply to CNVs that harbour non-coding RNA genes.

## Methods

### Study ethics approval

The study was approved by the local Health Research Ethics Board of Alberta (HREBA) - Cancer Committee.Written informed consents were obtained from all study participants. All experiments performed using specimens from study samples were carried out under approved guidelines and regulation.

### Study population

Women with confirmed diagnosis of invasive breast cancer (cases, n = 422) were recruited from Alberta, Canada between 1987 to 2006^51,56^, and were described earlier. Briefly, the cases were non-metastatic at the time of diagnosis. Median age at diagnosis was 52 years, and 90% of cases were diagnosed at age > 40 years (late age at onset); these are referred to as sporadic cases. Germline DNA and the clinical pathological information was accessed from the provincial tumor bank, the Alberta Cancer Research Biobank (formerly Canadian Breast Cancer Foundation (CBCF) Tumor Bank), located at the Cross-Cancer Institute, Edmonton, Alberta, Canada (http://www.acrb.ca/about-us/). At the time of study completion, the median follow-up time was 8.96 years and the number of events of breast cancer recurrence and death were n = 171 and n = 150, respectively. The controls (n = 348) were healthy women (median age 50 years) with no personal or family history of cancer at the time of recruitment. The controls were accessed from a prospective cohort study called the Tomorrow Project ((http://in4tomorrow.ca) from Alberta, Canada. Comprehensive information about study participants (cases and controls) and methods to extract germline DNA from buffy coats are described elsewhere^56,79^.

### Genotyping and Quality control

DNA extracted from buffy coat samples were genotyped using Affymetrix Genome-Wide Human SNP 6.0 array following manufacture’s protocol^56^. Affymetrix SNP 6 array has independent probes for SNPs (~ 906,600 probes) and CNVs (~ 946,000 probes). Genotyping quality control was assessed using Birdseed V2 algorithm in Affymetrix genotyping console. Sample Contrast Quality Control (CQC) ≥ 1.7 indicates acceptable genotyping quality. All our study samples had a CQC value more than 2.

### Population stratification

Principle Component Analysis (PCA) using EIGENSTRAT algorithm implemented in Golden Helix SNP and Variation suite v8.5.0 uses SNP genotypes generated on study samples (n = 762) to infer the population stratification. Genotype data from 270 HapMap samples were used as a reference to infer the genetic ancestry of the study samples, and these were described previously^56,57^. After removing the outlier samples, we had 366 cases and 320 controls classified as European ancestry, and these were used for copy number analysis.

We also carried out Identity by Descent (IBD) analysis based on SNP probes using Golden Helix SNP and Variation suite v8.5.0. These analyses did not reveal any cryptic relatedness in samples with pair-wise correlation cut off < 0.25.

### Copy number detection and gene annotation

Study design is described in Fig. 1. Copy Number Analysis was performed using Partek® Genomics Suite™ 6.6 (PGS). Affymetrix array generated CEL files were used as input files for the program. GC wave correction was applied using default functions. We created a reference baseline (all sample normalization) using all the study samples to assign a diploid status and to infer the relative copy number estimates in individual cases and controls. Genomic segmentation algorithm implemented in the software was used to call the genomic segments with the following default criteria: genomic markers > 10; P-value threshold = 0.001; Signal/Noise (S/N) ratio = 0.3. The copy number status was assigned for each inferred segment relative to the normalised intensity (*i*.*e*., 1.7–2.3 was considered as diploid); intensity values of > 2.3 and < 1.7 were called copy gains and losses, respectively. The CNVs were annotated using RefSeq genes using human genome build Hg19 (GRCh 37). The CNVs occurring at a frequency of > 10% (termed common CNVs) of the study samples and mapping (or overlapping) to the protein coding gene regions were considered for downstream analysis. We excluded the regions that mapped to small and long non-coding RNA genes and pseudogenes. Multiple CNVs with contiguous genomic break points and similar copy status in a genomic region were merged into a single Copy Number Variation Region (CNVR).

### Mapping to publicly available CNV databases

The identified CNVs were mapped to the Database for Genomic Variants^80^ (DGV, to ascertain CNVs calls). The structural variant data currently available through 1000 Genomes Project phase 3 has information about 60,000 structural variations captured at the population level. The project utilized low coverage whole genome sequencing and exome sequencing and microarray technologies. These germline datasets were utilized to compare the break points estimated for CNVs in our study and for potential overlap with coding genes^81^.

### Statistical Analysis

(i) Power calculations: Power to detect CNVs associated with Breast cancer susceptibility was calculated with “gap” package^82,83^ using R program^84^.  We estimate that the study design and the sample size used will confer 94% power to detect associations for breast cancer risk. The following assumptions were made to compute power with a sample size of n = 770: an additive model for genetic inheritance, the lifetime risk for breast cancer is 11% (1 in 9 among Caucasians) and at a genotype relative risk of 2 and a risk allele frequency of 10%.
(ii) Association analysis: The association frequencies of the CNVs (diploid, gain and loss) between sample categories (cases, controls) were compared using chi-square (2 × 3) test implemented in Partek® Genomics Suite™ 6.6. A multiple hypothesis testing was accounted for using a false discovery rate method (reported as q-value). CNVs were considered significant if q-values were < 0.05.
(iii) Survival analysis and Cox-proportional hazards model: CNVRs significantly associated with breast cancer risk by chi-square test were assessed for their prognostic significance of overall survival (OS) and recurrence free survival (RFS) using Cox-proportional hazards model, estimating Hazards Ratios (HRs) by the copy number status (diploid vs. gain/loss). Differences in survival probabilities among cases by the copy status (diploid vs gain/loss) were described using Kaplan-Meier survival curves. Survival analysis and Cox proportional hazards model were performed using “KMsurv” and “survival”^85,86^ packages, respectively, implemented in R^84^. Since only breast cancer associated CNVs with overlap to coding genes (n = 200 CNVs/CNVRs) and corrected for false discovery (q-value < 0.05) were considered for Cox analysis, we did not apply additional multiple hypothesis corrections.

### TaqMan copy number assays for validation of CNVs

CNVs were validated using TaqMan copy number assays from Applied Biosystems. Copy caller software supplied from Applied Biosystems was used for the data analysis. Representative CNVs were selected from three genes. We used predesigned assays for APOBEC3B (Hs04504055_cn), GSTM1 (Hs00273142_cn) and a custom assay for FGFR2 gene (assay location, chr10:123346308). Selection of genes for validation was based on the frequency of CNVs in our study cohort, availability of DNA in the corresponding samples with the inferred copy status for each sample from the copy number analysis. APOBE3B^47^ and GSTM1 loci^87^ were previously characterized to show copy number deletions. We used RNAase P as an internal control and followed the manufacturer-supplied protocols. We used two genomic DNA specimens from the Coriell DNA panel as positive controls. NA18635, which is of Chinese ancestry and diploid for all three genes tested, was used for data normalization. NA05299 belongs to European ancestry and has deletion in FGFR2 region.

### Gene expression (mRNA) analysis in breast tumor tissues

mRNA dataset (Gene expression dataset) generated on breast tumor samples using Agilent Whole Human Genome Microarray 4 × 44 K (GEO Accession ID: GSE22820) was available in-house with patient clinical characteristics (n = 90). The 90 breast cancer cases were a subset of 366 (PCA stratified) cases with copy number profiles. Raw intensity files were quantile normalized, and log2 transformed using Partek Genomics Suite v6.6. The linear correlation was estimated between the germline copy number status and gene expression using PGS algorithms. In the correlation analysis, we considered only those gene expression probes whose location is within the breakpoints of the CNVs interrogated.

The objectives were to characterize the gene dosage effects and the relative expression of CNV-genes in breast tissues: (i) The dosage sensitive genes were determined by Pearson’s correlation analysis (using PGS) between copy number and gene expression, and correlation value r > 0.20. For the significantly correlated CNVs, dot plots of breast tumor gene expression versus germline copy number status were plotted. (ii) The prognostic significance of the genes overlapping in the germline CNV-genes from RFS and OS were also examined for breast tumor tissue specific gene expression. Fifteen of the 16 genes overlapping in the CNVR associated with OS were expressed. For ten genes in CNVR associated with RFS, eight genes were expressed in the mRNA dataset. Considering these genes as continuous variables, Univariate Cox proportional hazards regression was performed using SPSS v21.

### Availability of data and material

All data generated or analysed during this study are included in this published article and its supplementary information files.

## Electronic supplementary material

Supplementary dataset 1
